# Analysis of Clinical Symptoms of Guillain–Barré Syndrome Induced by Heat Stroke: Three Case Reports and Literature Review

**DOI:** 10.3389/fneur.2022.910596

**Published:** 2022-06-17

**Authors:** Xiao-xiao Ni, Cong-lin Wang, Ye-qun Guo, Zhi-feng Liu

**Affiliations:** ^1^Department of Hyperbaric Oxygen Medicine and Rehabilitation, General Hospital of Southern Theater Command of PLA, Guangzhou, China; ^2^Department of Critical Care Medicine, General Hospital of Southern Theater Command of PLA, Guangzhou, China

**Keywords:** heat stroke, nervous injury, Guillain-Barré syndrome, peripheral neuropathy, nervous system

## Abstract

**Background:**

Heat stroke is a potentially fatal condition that is caused by elevated core temperature. Guillain–Barré syndrome (GBS) induced by heat stroke is extremely rare and has only been reported in few case reports. The purpose of this case study was to evaluate the clinical symptoms, neuroelectrophysiological and imageological features of GBS after heat stroke.

**Methods:**

We reviewed our hospital records and previously published reports to find the cases of GBS after heat stroke. The clinical, imageological, and electrophysiological profiles, treatment and prognosis were presented and analyzed.

**Results:**

We retrieved three cases of GBS induced by heat stroke from our hospital, which presented as lesions on multiple cranial and peripheral nerves and albuminocytologic dissociation in the cerebrospinal fluid. All of these patients had disorders of consciousness at the early stage of heat stroke and a “pseudo-recovery period” after they recovered from coma after heat stroke. After immunoglobulin administration and immunoregulation therapy, these patients' neurological deficiencies were relieved significantly. But there are still disabilities and almost totally reliant on others.

**Conclusions:**

The number of the cases of GBS induced by HS reported in this study has been the most in the recent 5 years. Clinicians should pay attention to patients with heat stroke with sustained coma and the sudden quadriplegia. Early, exact and timely diagnosis and treatment of GBS need to be performed, to accelerate recovery and improve prognosis.

## Introduction

Heat stroke (HS) is a life-threatening disease and the most severe condition of heat-related illness, characterized by core body temperature >104°F (40°C) and neurological dysfunction such as confusion, seizures, or loss of consciousness (1). HS is divided into nonexertional HS and environmental heat stroke (EHS). The central nervous system is highly sensitive to hyperthermia, leading to neurological complications due to the involvement of the cerebellum, basal ganglia, and peripheral nerves. There are various manifestations of nervous system injuries after HS, including cerebellar injury, subarachnoid hemorrhage, peripheral nerve injury, and so on (2). However, what is the relationship of HS and Guillain–Barré syndrome (GBS)? Relevant reports are few and GBS after HS was sometimes thought to be unrelated to HS. The exact relationship between GBS and HS remains to be studied.

Guillain–Barré syndrome is considered to be the most common cause of acute flaccid paralysis that affects all age groups worldwide and can lead to disability and high risk of mortality (3). Most patients present with an antecedent illness, most commonly upper respiratory tract infection, before the onset of progressive motor weakness (4). Heat stroke as an inducement of GBS was observed in the recent years. GBS is a group of acute immune-mediated paralytic neuropathies characterized by rapidly progressive bilateral weakness of the extremities with hyporeflexia or areflexia (5). Subtypes are mainly categorized into demyelinating and axonal forms according to the clinical course, nerve conduction velocities, and immunologic findings. The typical form of GBS is acute inflammatory demyelinating polyradiculoneuropathy (AIDP), whereas the main axonal forms are subdivided into acute motor axonal neuropathy (AMAN) and acute motor and sensory axonal neuropathy (AMSAN) (3). The pathological changes are lymphocyte infiltration and macrophage infiltration around small and medium blood vessels of peripheral nerve tissue and nerve fiber demyelination. The development of GBS is associated with the phenomenon of molecular mimicry and with cross-reactivity. At present, it is believed that GBS is an autoimmune disease, in which the body's immune system produces immune responses against peripheral nerves, resulting in peripheral nerve demyelination (3). GBS after HS is very easily misidentified with peripheral nerve injury. How to distinguish GBS from peripheral nerve injury after HS is a clinical problem?

In the past 5 years, we have retrieved 3 cases of acute GBS induced by HS in our hospital. In general, GBS after heat stroke has only been reported in case reports, and there are few reports describing the findings on clinical features of GBS after HS. Therefore, this study aims to observe the clinical progress of GBS after HS, changes in immunological indicators and the efficacy of immunoregulatory therapy.

## Materials and Methods

This study was conducted in accordance with the Declaration of Helsinki. We reviewed the electronic medical records for patients who had HS from 1 January 2016 to 1 March 2022 in our hospital. For all cases from our hospital records, we recorded the patient's gender, age, blood pressure, core temperature (immediately after admission), Glasgow coma scale (GCS) on arrival, DIC score, liver and kidney function, blood coagulation function, blood routine, cardiac indicators, analysis of cerebrospinal fluid (CSF), results of neuroelectrophysiological examination, brain magnetic resonance imaging (MRI), and the clinical prognosis. The same variables were collected from the previously reported cases if available (clinical background, patient characteristics, results of neuroelectrophysiological and laboratory examination, MRI findings, and prognosis). The Barthel index was evaluated to assess daily living ability at 90 days after HS.

For literature research, we reviewed published scientific reports by searching the PubMed database and China National Knowledge Infrastructure (CNKI). Search strategies were developed to find publications containing subject headings and key words (text words) including heat stroke, heat or sun disorder, injury, illness, stress, related, shock, exhaustion; heat stroke; neurology; nervous system; Guillain–Barré syndrome; peripheral neuropathy; and case, cases. Papers included were limited to human subjects and publication types (case reports, case series). Publications of cases that had an exertional component or nonexertional HS (e.g., confined spaces, Bikram Yoga, and electric blankets) were both included in this study. Papers excluded from review were those unrelated to Guillain–Barré syndrome or peripheral neuropathy or where individual patient data were not available.

## Results

**Figure 1:**
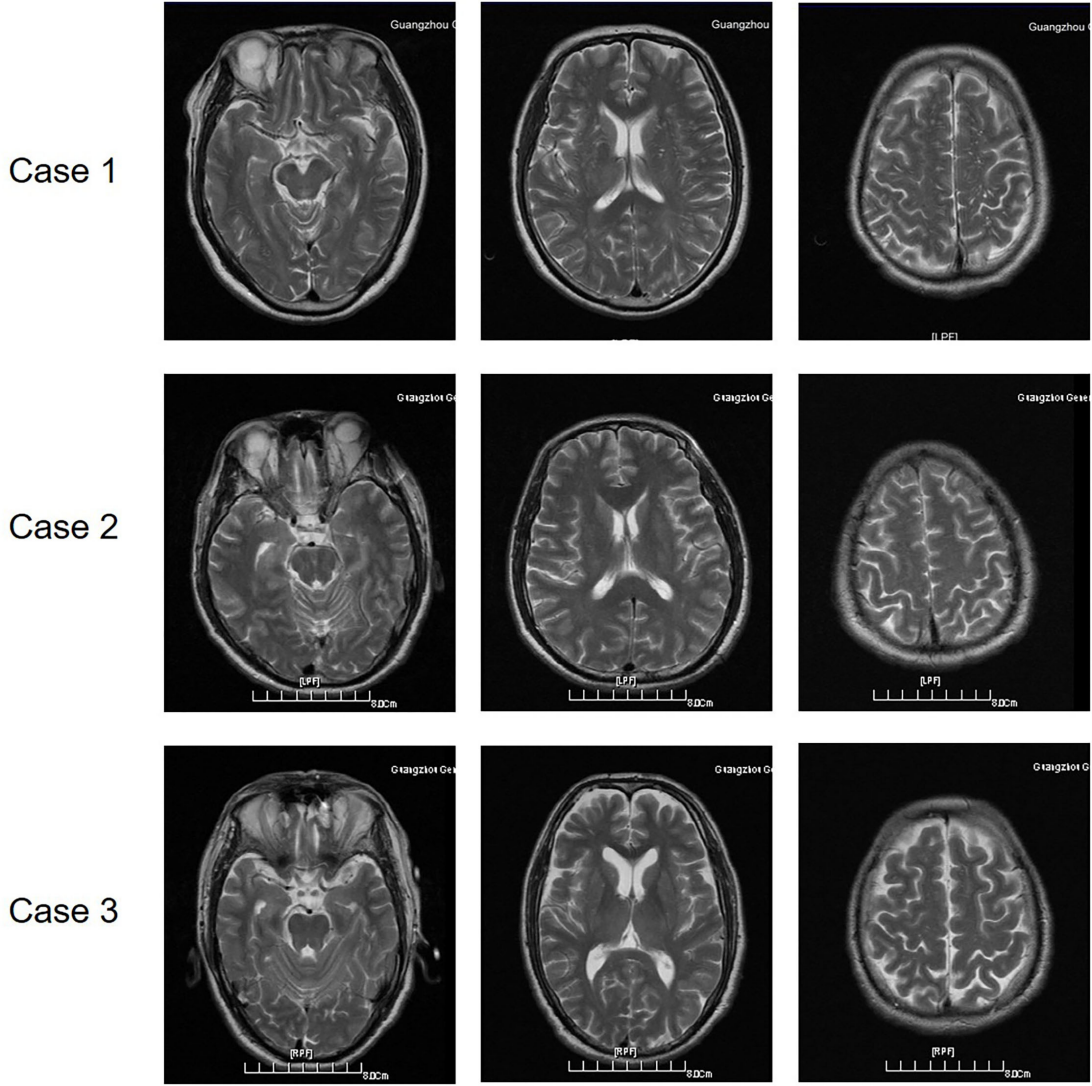
The brain magnetic resonance imaging (MRI) of the three patients with GBS induced by HS.

**Figure 2:**
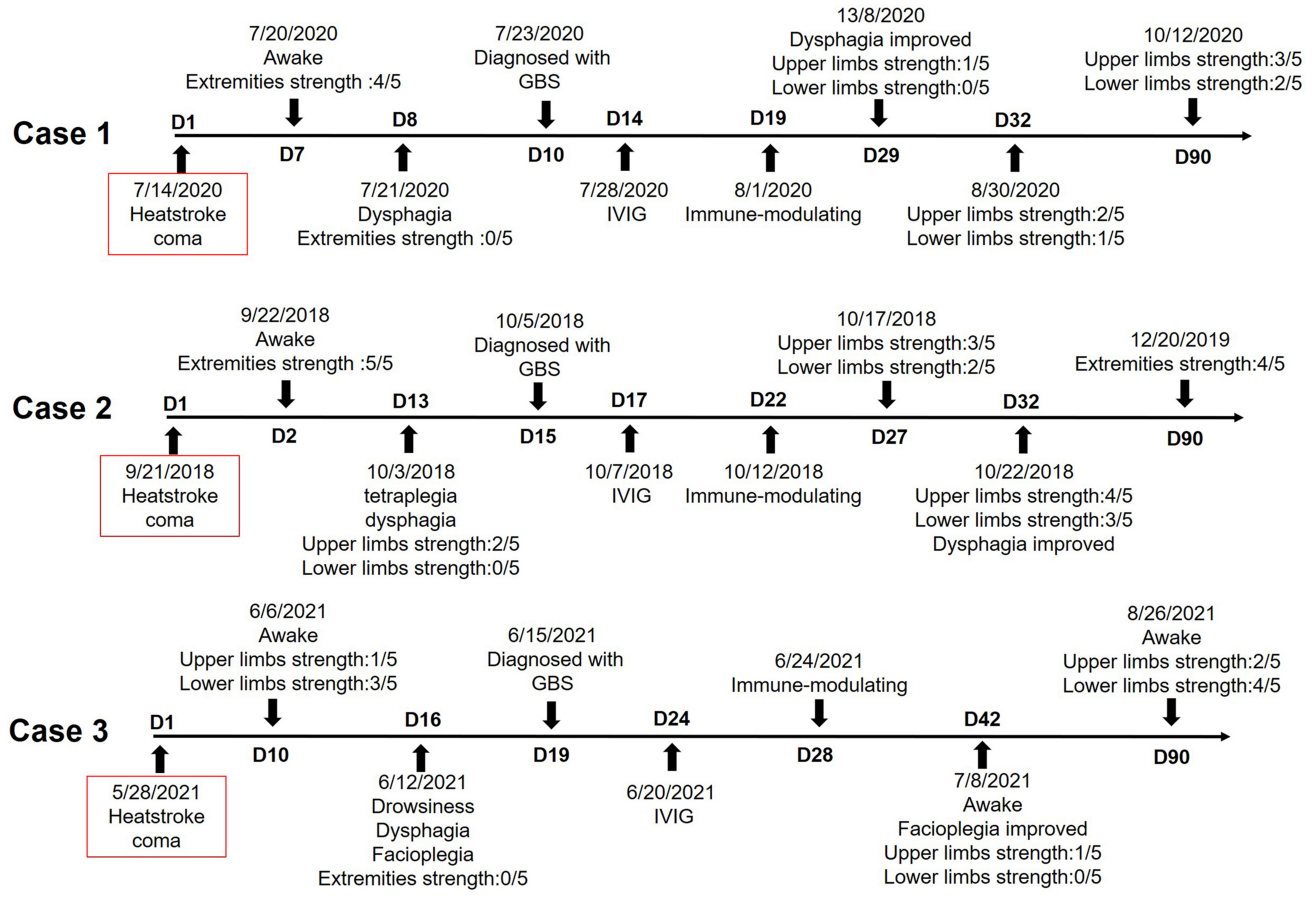
The timeline of the disease and treatment of these three patients with GBS induced by HS.

A total of 6,286 publications (3,379 from PubMed, 2,907 from CNKI) between 1990 and 2021 were identified using the structured online search strategy. A total of 48 articles were screened in full-text review and 6,238 publications were excluded. A total of three articles were unable to obtain and 37 articles were not clinically relevant. A total of four articles including 4 cases were included following review (6–9). We extracted 3 cases from our hospital (searching between January 2016 and March 2022). The detailed clinical data of these seven cases are shown in Table 1. The average age was 40.4 ± 4.73 years (range, 25–57 years), and most of the patients were men (85.7%). About 71.43% (5/7) of the patients had multiple organ damage (MOD). The average GCS score was 5.33 points (range, 3–10 points) and the duration of coma was 5.5 ± 0.71 days. The average duration from HS to extremity weakness was 13.2 ±1.31 days (range, 8–15 days). There was a period from the patient recovering from coma to developing quadriplegia, which was called “pseudo-recovery period” by us. The average duration of “pseudo-recovery period” was 7.6 ± 1.67 days (range, 1–12 days). Albuminocytologic dissociation (protein cell separation) was shown in the cerebrospinal fluid (CSF) collected by lumbar puncture of all these patients. Electromyography of these patients revealed impairment of sensorimotor fibers in bilateral median, ulnar, tibial, and common peroneal nerves. MRI of these patients revealed no abnormality or a small amount of ischemic focus (Figure 1). About 28.57% (2/7) of the patients had abnormal antibodies in CSF, and all of them responded to immunomodulatory therapy. Neurological sequelae continued up to 90 days after HS and the Barthel index of these patients was 38.75 ± 20.9 (range, 10–100). The conduction velocity of sensorimotor nerves of the three patients in our hospital is shown in Table 2. Figure 2 shows the timeline with relevant data from the episode of care of these three patients.

Table 1Detailed clinical data of patients with Guillain–Barre syndrome induced by heat stroke.

**Table:** 

**No**	**Source**	**Year**	**Location**	**Background Diseases**	**Age**	**Gender**	**Tc (**°**C)**	**Degree of coma**	**Duration of coma (day)**	**GCS scale**
1	Our hospital	2020	China	Hypertension	57	Male	42	deep	7	3
2	Our hospital	2018	China	None	41	Male	42.5	light	6	10
3	Our hospital	2021	China	None	56	Male	42	deep	8	3
4	J Neurol Neurosurg Psychiatry, 1999, 66(3):408.	1999	Germany	Drug addict	28	Male	42.5	deep	4	NA
5	Clin Lab, 2017, 63(9):1507-1511.	2017	China	NA	41	male	41.3	moderate	5	NA
6	J Environ Occup Med (China), 2007, 24(3):325-326	2007	China	None	35	male	42	Deep	NA	NA
7	J Binzhou Med College (China), 1998, 21(6):562	1998	China	NA	25	female	39.8	deep	3	NA

**Table:** 

**No**	**BP (mmHg)**	**MOD** **(Yes/No)**	**HS to quadriplegia (day)**	**Pseudo-recovery period (day)**	**Cranial nerve injury**	**Both upper limbs strength (MRC grades)**	**Both lower limbs strength (MRC grades)**
1	95/43	Yes	8	1	Dysphagia, facioplegia, dysarthria	0	0
2	92/46	Yes	13	11	Dysphagia, facioplegia, dysarthria	2~3	0
3	87/36	Yes	16	6	Dysphagia, facioplegia, ophthalmoplegia	0	0
4	85/30	Yes	12	8	Dysphagia, facioplegia	2~3	1~2
5	NA	NA	NA	NA	Dysphagia, right blepharoptosis, dysarthria	2	2
6	141/85	Yes	15	NA	Dysphagia, dysarthria	0	0
7	NA	NA	15	12	facioplegia	0~1	0~1

**Table:** 

**No**	**Protein CONC in CSF (g/l)**	**WBC in CSF (10** ^**6**^ **/L/mm** ^**3**^ **)**	**Antibody in CSF**	**Treatment**	**Prognosis/time**	**The Barthel index**
1	0.47~1.82	0.6	None	Plasma exchange; IVIG; Immune-modulating.	Upper limb strength (MRC grades 3/5); Lower limb strength (MRC grades 2/5); /day 90	10
2	0.62~0.88	3	IgG (59 mg/L) IgA (2.55 mg/L)	IVIG; Immune-modulating.	Extremities strength (MRC grades 4/5); /day 90	30
3	1.09	3.4	None	IVIG; Immune-modulating.	Upper limb strength (MRC grades 2/5); Lower limb strength (MRC grades 4/5); /day 90	15
4	0.48~7.3	6	IgM (500 U/l)	IVIG; plasma exchange.	Chair-bound /14 months later	NA
5	0.732	6	NA	IVIG; Immune-modulating.	Recovered /3 months later	100
6	1.005	NA	NA	Immune-modulating.	Extremity muscles weakness /NA.	NA
7	2.85	3	NA	Immune-modulating.	Extremity strength (MRC grades 3/5) /day 35	NA

*MOD, multiple organ damage; BP, blood pressure; MRC, Medical Research Council; CONC, concentration; IVIG, intravenous immunoglobulin*.

**Table 2:** The conduction velocity of nerves.

	**Nerve**	**Sensory nerve**			**Motor nerves**		
		**Latency (ms)**	**Amplitude (μV)**	**Conduction velocity (m/s)**	**Latency (ms)**	**Amplitude (mV)**	**Conduction velocity (m/s)**
Case 1	Right median nerve	3.52	1.95	47.9	8.35	0.45	46.6
	Left median nerve	3.49	1.71	48.1	8.38	0.58	46.3
	Right ulnar nerve	3.02	2.2	48.7	6.67	0.69	53.7
	Left ulnar nerve	0	0	0	6.65	0.87	54.2
	Right tibial nerve	0	0	0	13	0.61	40.5
	Left tibial nerve	0	0	0	12.9	0.83	41
	Right common peroneal nerve	0	0	0	10.5	0.43	45.6
	Left common peroneal nerve	0	0	0	10.5	0.43	45.4
Case 2	Right median nerve	3.23	5.9	55.1	9	1.42	46
	Left median nerve	3.21	4.9	55.1	8.78	1.42	46.4
	Right ulnar nerve	2.63	6.6	51.6	7.45	1.02	48.3
	Left ulnar nerve	2.69	4.5	51.2	7.03	3.6	49.2
	Right tibial nerve	0	0	0	20.4	0.48	33.6
	Left tibial nerve	0	0	0	20	0.62	34.4
	Right common peroneal nerve	0	0	0	14.7	0.51	36.9
	Left common peroneal nerve	0	0	0	14.5	0.39	36.5
Case 3	Right median nerve	3.45	1.75	43.1	12.35	0.43	37.8
	Left median nerve	3.03	1.75	41.5	15.31	0.51	40.1
	Right ulnar nerve	3.56	1.85	45.3	10.7	0.52	42.3
	Left ulnar nerve	3.58	1.80	42.9	9.81	0.56	41.6
	Right tibial nerve	0	0	0	12.08	0.35	37.8
	Left tibial nerve	0	0	0	13.73	0.39	35.5
	Right common peroneal nerve	0	0	0	8.76	0.40	43.3
	Left common peroneal nerve	0	0	0	12.58	0.38	40.9

*The conduction velocity of motor nerves of median nerve and ulnar nerve was recorded through the stimulation from elbow to wrist. The conduction velocity of motor nerves of tibial nerve and common peroneal nerve was recorded through the stimulation from knee to ankle*.

## Case Reports

### Case 1

A 57-year-old man was found unresponsive in a workshop on a hot summer day (ambient temperature 36°C). His core temperature was 42°C within 30 min of admission. He was in deep coma with wide unreactive pupils and without corneal and pharyngeal reflexes. Blood pressure was 95/43 mmHg and the heart rate was 146/min. He developed disseminated intravascular coagulation (DIC), thrombopenia below 1,800 MRD/ml, acute heart/liver/kidney damage, rhabdomyolysis, and metabolic acidosis. He was diagnosed with HS. After 7 days of treatment including hyperbaric oxygen (HBO), correction of electrolyte and acid-base disturbance, antishock, protection of cardiac and hepatorenal function, suppression of excessive gastric acid secretion, etc., his consciousness recovered from coma and his limb muscles had Medical Research Council (MRC) grades 4. But only 1 day later, he complained of fatigue, weakness of limbs, dyspnea, and difficulty in expectoration. He required intensive care and tracheotomy within hours. All limb muscles had MRC grade 0. Facioplegia increased 2 days later. He had no ophthalmoplegia, but having hyporeflexia and stocking glove hypoesthesia for vibratory and cold stimuli. Small ischemic focus in the basal ganglia and cortex was shown through MRI. Protein concentration in CSF was 0.47 g/l at 2 days after tetra paresis and 1.82 g/l 10 days later. White blood cell (WBC) count was 0.60 × 10^6^/L cells/mm^3^ at 2 days after tetra paresis and 5.4 × 10^6^/L cells/mm^3^ 10 days later. Antibody concentration in the CSF and serum was all normal. Compound motor action potentials were <1 mV in all tested nerves at 10 days after HS. Conduction velocities were below 90% of the lower limit of normal in the bilateral tibial nerve and common peroneal nerves. F responses were missing in both median nerves, ulnar nerves, tibial nerves, and common peroneal nerves. A conduction block was present along the both median nerves (right wrist stimulation amplitude 0.67 mV and left 0.56 mV; right elbow stimulation amplitude 0.45 mV and left 0.58 mV) and ulnar nerves (right wrist stimulation amplitude 0.76 mV and left 0.88 mV; right elbow stimulation amplitude 0.69 mV and left 0.87 mV). The sensory potentials of bilateral tibial nerves and common peroneal nerves were not elicited. The amplitude of the sensory potentials of bilateral median nerves and ulnar nerves was reduced. The patient was diagnosed with AIDP and treated with a 2-day course plasma exchange, intravenous immunoglobulin (IVIG) for 5 days, and then immune-modulating therapy (methylprednisolone). The strength of both upper limbs recovered to MRC grade 1, and the patient was discharged from hospital 29 days after HS. Then, 3 months after HS, upper limb muscles had MRC grade 3 and lower limb muscles had MRC grade 2, which made the patient chair-bound.

### Case 2

A 41-year-old man fainted suddenly with projectile vomiting while working on a hot summer day (ambient temperature 35°C). His core temperature was 42.3°C on admission. He was in light coma with corneal, pharyngeal, and pupillary light reflexes. Tachypnoea had induced hypocapnia. Blood pressure was 92/46 mm Hg and the heart rate was 165/min. He had hepatic and renal dysfunction, inflammatory reaction, myocardial ischemia, and coagulation disorders. No abnormalities were found in brain MRI and he was diagnosed with HS. After 2 days of coma, he was conscious and the MRC grades of his limbs were level 5 (normal). But after 8 more days, he complained of fatigue, myalgia, and arthralgia, and 3 days later, he developed fever (38.9°C), tetraplegia, and dysphagia. He required intensive care within hours. Proximal arm muscles had MRC grades between 2 and 3. All other limb muscles had grade 0 and he developed facioplegia. Protein concentration in CSF was 0.88 g/l and WBC count was 3 × 10^6^/L at 1 day after the onset of tetra paresis. Protein concentration in CSF was 0.62 g/l and WBC count was 1.3 × 10^6^/L at after 2 more days. The concentration of IgG antibody in the CSF was 59 mg/L, and concentration of IgA antibody was 2.55 mg/L at 2 days after the onset of tetra paresis. Compound motor action potentials were <1.5 mV in all tested nerves 14 days after HS. Conduction velocities were below 90% of the lower limit of normal in the bilateral tibial nerves and common peroneal nerves. F responses were missing in both tibial nerves and common peroneal nerves. F responses were decreased in both median nerves and were normal in both ulnar nerves. A conduction block was present along the both median nerves (right wrist stimulation amplitude 1.85 mV and left 1.86 mV; right elbow stimulation amplitude 1.42 mV and left 1.42 mV) and tibial nerves (right ankle stimulation amplitude 0.42 mV and left 0.46 μV; right knee stimulation amplitude 0.48 mV and left 0.62 mV). The sensory potentials of the bilateral tibial nerves and common peroneal nerves were not elicited. The patient was diagnosed with AIDP and treated with IVIG for 5 days and then immune-modulating therapy (methylprednisolone). The strength of the limbs recovered to MRC grade 3, and the patient was recovered from facioplegia 32 days after HS. The patient was relieved from tracheotomy and could stand up with walking aid 90 days after HS.

### Case 3

A 56-year-old man felt dizziness and fatigue while working in a hot environment (ambient temperature 33°C). Immediately, the patient developed blurred consciousness, irritability, and progressed to coma and accompanied by great sweating. He was admitted to emergency room immediately. His core temperature was 42°C, with a blood pressure of 87/36 mm Hg and the pulse rate of 164/min. He was in a deep comatose state, hepatorenal dysfunction, hypokalemia, elevated inflammatory reaction, myocardial ischemia associated with increased myocardial enzymes, coagulation disorders, and rhabdomyolysis. No abnormalities were found in brain MRI. He was diagnosed with HS and was treated by cooling, antishock, and intubation. He was conscious after 10 days of coma. The strength of his upper limbs had MRC grade 1 and the lower limb muscles had grade 3. His hepatorenal dysfunction, inflammatory reaction, myocardial ischemia, and coagulation disorders were alleviated after 12 days. But after 6 more days, the patient developed drowsiness, tetraplegia with MRC grade 0 of the muscle strength, dys-expectoration, and required tracheotomy. Protein concentration in CSF was 1.09 g/l and WBC count was 3.4 × 10^6^/L at 3 days after tetra paresis. Immunoglobulin in CSF and serum was normal. Compound motor action potentials were <1 mV in all tested nerves at day 18. Conduction velocities were below 90% of the low limit of the normal value in both median nerves and ulnar nerves. F responses were missing in tibial nerves, ulnar nerves, and common peroneal nerves. A conduction block was present along the both median nerves (right wrist stimulation amplitude 0.78 mV and left 0.56 μV; right elbow stimulation amplitude 0.43 mV and left 0.51 mV) and tibial nerves (right ankle stimulation amplitude 0.53 mV and left 0.55 mV; right knee stimulation amplitude 0.35 mV and left 0.39 mV). The sensory potentials of the bilateral tibial nerves, ulnar nerves, median nerves, and common peroneal nerves were not elicited. The patient was diagnosed with AIDP at 19 days after HS and treated with a 5-day course IVIG and then immune-modulating therapy (methylprednisolone). He was sober 21 days after HS and his upper limbs had MRC grade 1 at 42 days after HS. His upper limb strength had MRC grade 2 and lower limb strength had MRC grade 4 at 90 days after HS.

## Discussion

These patients in our hospital developed an acute neuropathy that met clinical and neurophysiological criteria for Guillain–Barré syndrome. Currently, similar reports are very rare, but we have treated three cases of such disease in the recent 5 years. Neurological dysfunction is an important clinical feature of heat stroke. Patients with cerebellar injury account for the majority of neurological sequelae induced by HS (10). GBS-like neuropathies have been reported from Saudi Arabia, where the temperature is high and HS is common (11). In this study, 85.7% (6/7) of the cases were from China. GBS symptoms developed almost 10 days after HS. The patients with GBS all had consciousness disorders at the early stage of HS and limb weakness often developed after patients regained consciousness. Moreover, patients with mild degree of disorder of consciousness had better prognosis. The mechanism may be related to cerebral hypoxia and secondary neuron death induced by HS.

Different from other peripheral nerve injuries induced by HS, there was a “pseudo-recovery period” after these patients recovered from coma after HS. In the “pseudo-recovery period,” the extremity sensorimotor function of these patients was improved. Tetraplegia and dysphagia usually developed after the “pseudo-recovery period.” Protein cell separation was shown in the CSF of all these patients, which is a typical characteristic of GBS. The results of electromyography and clinical presentation supported the diagnosis of GBS. GBS is an acute polyradiculoneuropathy that typically presents with progressive monophasic generalized and symmetric weakness classically involving bilateral upper and lower extremities and associated with hyporeflexia. A total of three patients in this study were diagnosed with AIDP. AIDP is the most common GBS variant, with electrophysiological studies primarily showing demyelination and variants showing primary dysfunction or loss of peripheral nervous system axons (12). The immunological cascade that may trigger and induce demyelination in peripheral nerves in AIDP patients is complex and incompletely understood (12). Heat stroke and its progression to nervous system injury are due to a complex interplay among the acute physiological alterations associated with hyperthermia (including circulatory failure and hypoxia), the direct cytotoxicity of heat, cell apoptosis, and the inflammatory and coagulation responses of the host. The mechanism of AIDP after HS may be related to demyelination induced by hyperthermia. Axonal excitability can be reduced, and conduction block of motor and sensory axons may occur during hyperthermia (13). However, the particular mechanism of demyelination induced by HS is still less studied.

Compared with classical presentation of GBS, the disease progressed rapidly and relieved within 14 to 26 days of onset. But the prognosis was poor and almost totally reliant on others. Sural sparing pattern is a typical symptom of GBS, which strongly supports the diagnosis of GBS in half to two-thirds of patients (14, 15). About 38% of patients with AIDP showed sensory nerve conduction abnormalities in the sural nerves (16). A total of three patients in our hospital had abnormal sensory nerve conduction of common peroneal and tibial nerves, and sural nerve conduction was moderately slowed in Case 4. It suggested that peripheral nerve injury induced by GBS after HS might be more serious than classical GBS. Although the sural-sparing pattern has not yet been included in the electrodiagnostic criteria, it plays a well-established role in GBS diagnosis (17). The lack of sural nerve examination was a limitation of this study. These patients were treated with methylprednisolone following plasma exchange or IVIG. Although corticosteroids given alone do not significantly hasten recovery from GBS (18), intravenous methylprednisolone combination with IVIG may hasten recovery but does not significantly affect the long-term outcome (19). In this study, the symptoms of these three patients improved significantly after treatment, suggesting the effect of combination therapy of GBS after HS.

Acute inflammatory demyelinating polyradiculoneuropathy has a relatively better prognosis than axonal forms of GBS. In this study, the MRC grades of extremities strength of these patients were increased 90 days after HS, and cranial nerve disturbances were relieved. But they were still chair-bound. Therefore, GBS should be identified, diagnosed, and treated early when the patient develops extremity sensorimotor disturbances after regaining consciousness from HS.

## Conclusion

To our knowledge, the most cases of GBS induced by HS have been reported in this study in the recent 5 years. Clinicians should pay attention to heat stroke patients with sustained coma. When extremity weakness, tetraplegia, and dysphagia develop after the patient recover consciousness, early, exact, and timely diagnosis and treatment should be performed, to accelerate recovery and improve prognosis.

## Data Availability Statement

The original contributions presented in the study are included in the article/supplementary material, further inquiries can be directed to the corresponding author.

## Ethics Statement

Written informed consent was obtained from the individual(s) for the publication of any potentially identifiable images or data included in this article.

## Author Contributions

XXN: data curation and writing-original draft preparation. CLW: data curation. YQG: methodology. ZFL: writing and reviewing and editing. All authors contributed to the article and approved the submitted version.

## Funding

This work was supported by grants from the National Natural Science Foundation of China [No. 82072143], Natural Science Foundation of Guangdong Province of China [No. 2021A1515010170], the PLA Logistics Research Project of China [18CXZ030, BLJ20J006], and the PLA Medical Science and Technology Youth Development Programme [21QNPY122].

## Conflict of Interest

The authors declare that the research was conducted in the absence of any commercial or financial relationships that could be construed as a potential conflict of interest.

## Publisher's Note

All claims expressed in this article are solely those of the authors and do not necessarily represent those of their affiliated organizations, or those of the publisher, the editors and the reviewers. Any product that may be evaluated in this article, or claim that may be made by its manufacturer, is not guaranteed or endorsed by the publisher.
